# Identification of Novel Environmental Substances Relevant to Pediatric Graves’ Disease

**DOI:** 10.3389/fendo.2021.691326

**Published:** 2021-06-23

**Authors:** Qin Xia, Jingjing Liu, Xu Xu, Wei Gu, Kefeng Gu, Xiuli Chen, Rongrong Xie, Dandan Zhang, Haiying Wu, Hui Sun, Fengyun Wang, Linqi Chen, Ting Chen

**Affiliations:** ^1^ Department of Endocrinology, Genetics and Metabolism, Children’s Hospital of Soochow University, Suzhou, China; ^2^ Department of Biochemistry and Molecular Biology, School of Medical and Biological Sciences, Soochow University, Suzhou, China; ^3^ Department of Pediatric Endocrinology, The Affiliated Wuxi Children’s Hospital of Nanjing Medical University, Wuxi, China; ^4^ Department of Endocrinology, Children’s Hospital Affiliated to Nanjing Medical University, Nanjing, China

**Keywords:** Graves’ disease, children, metabolomics, environmental substances, single-nucleotide polymorphisms

## Abstract

Graves’ disease (GD) is the most common cause of hyperthyroidism, yet a relatively rare disease in the pediatric population. GD is a complex disorder influenced by both genetic and environmental factors. In this study, we aimed to find new environmental factors influencing the pathogenesis of GD. We investigated serum substances in 30 newly diagnosed GD children and 30 age- and gender-matched healthy controls. We measured total iodine by inductively coupled plasma-mass spectrometry (ICP-MS), analyzed perfluorinated compounds *via* ultra-high-performance liquid chromatography coupled with multiple reaction monitoring mass spectrometry (UHPLC-MRM-MS), and explored other environmental substances using ultra-high-performance liquid chromatography–quadrupole time-of-flight mass spectrometry (UHPLC–QTOF/MS) analysis. Twenty-nine single-nucleotide polymorphisms (SNPs) in eight genes related to GD were analyzed by SNaPshot. The serum total iodine was significantly higher in GD group, but its association with GD onset was weak, only with Exp(B) value near 1. The perfluorinated compound levels were not different between the two groups. More importantly, we found 16 environmental substances significantly different between GD and control groups, among which ponasterone A is a risk factor (*p* = 0.007 and Exp(B) = 14.14), while confertifoline is a protective factor against GD onset (*p* = 0.002 and Exp(B) = 0.001). We also identified 10 substances correlated significantly with thyroid indices in GD patients, among which seven associated with levels of the thyroid autoantibody TPOAb. No known SNPs were found predisposing GD. In this study, we explored a broad variety of environmental substances and identified novel factors that are potentially involved in the pediatric GD pathogenesis.

## Introduction

Although relatively rare in childhood, Graves’ disease (GD) is the most common cause of hyperthyroidism in the pediatric population (1, 2). The incidence of GD in children is about 0.9–14.1/100,000, peaking in adolescence with a strong female-to-male predominance (3, 4). There is a worldwide trend of increasing incidence of juvenile thyrotoxicosis (3, 4), with two to three times higher incidence in Chinese children than that in the Caucasian (3, 4).

GD is a complex disease caused by a complex interplay among genetic and non-genetic factors, leading to sustained autoimmune response and loss of immune tolerance to thyroid antigens (5). Previous epidemiological researches on GD environmental factors have studied the impact of iodine, infectious diseases, psychological stress, smoking, vitamin D, selenium, and immune modulating agents on GD pathogenesis (5), among which inappropriate iodine supply plays a major role in eliciting the occurrence of GD in genetically predisposed individuals (6). Thyroid-disrupting chemicals (TDCs) are man-made chemicals that can disrupt the synthesis, circulating concentrations, and peripheral action of the thyroid hormone (7). Studies on TDCs have implied their roles in the pathogenesis of autoimmune thyroid disease (AITD) (8). Perfluorinated compounds are a group of recently reported TDCs proved to affect thyroid function in children (9, 10). Their relationship with GD has not yet been explored.

In the present study, we have recruited untreated GD children and their age- and gender-matched healthy controls and collected fasting serum samples from all participants. Then we measured serum total iodine by inductively coupled plasma-mass spectrometry (ICP-MS). We explored the serum levels of perfluorinated compounds *via* ultra-high-performance liquid chromatography coupled with multiple reaction monitoring mass spectrometry (UHPLC-MRM-MS). More importantly, we surveyed small molecule environmental substances using ultra-high-performance liquid chromatography–quadrupole time-of-flight mass spectrometry (UHPLC–QTOF/MS) analysis, in order to find new environmental factors affecting the GD pathogenesis. Besides, to investigate how the genetic factors interacting with the environment, we also analyzed 29 known single-nucleotide polymorphisms (SNPs) from eight genes most related to GD in the Asian population.

## Materials and Methods

### Study Design and Participants

This study was approved by the medical ethics committee of Children’s Hospital of Soochow University, and written informed consent was obtained from all participants’ parents, according to the guidelines of Declaration of Helsinki 2000. The Institutional Review Board approval was also obtained before the study. We collected blood samples of 30 untreated patients (25 girls and five boys) newly diagnosed with GD at Children’s Hospital of Soochow University between March 2017 and May 2018. Another 30 age- and gender-matched healthy subjects were enrolled from their annual physical examination as controls. All the participants are local citizens of Suzhou, a city in southeast China. The clinical characteristics of all the participants were summarized in **Table 1**. Before the onset of study, all the patients and control subjects had undertaken general physical examination and laboratory evaluation. Those patients with liver dysfunction, cardiovascular complications, or other endocrine disorders were excluded from the study. To avoid the impact of sex hormones, we evaluated pubertal development *via* Tanner staging. Only boys with testicular volume smaller than 4 ml and girls with breast development earlier than Tanner stage II were recruited in the study.

**Table 1:** Clinical characteristics of the GD and control groups.

	Normal range	GD group (n = 30)	Control group (n = 30)	*p* Value
Age (months) ^a^	NA	78.80 ± 20.50	72 ± 20.42	NS
Girls/Boys	NA	25/5	25/5	—
Wt (kg) ^b^	NA	24.86 (20.38–28.25)	26.48 (22.73–29.16)	NS
FT3 (pg/ml) ^a^	2.71–4.69	11.41 ± 6.0	3.91 ± 0.32	<0.01
FT4 (ng/dl) ^a^	1.04–1.83	4.33 ± 2.41	1.37 ± 0.16	<0.01
TSH(μIU/ml)^a^	0.91–4.63	0.0067 ± 0.0039	2.61 ± 1.31	<0.01
TT3 (ng/ml) ^a^	0.81–2.43	3.18 ± 1.45	1.12 ± 0.23	<0.01
TT4 (ng/ml) ^a^	55.33–124.22	157.92 ± 71.58	80.12 ± 13.33	<0.01
TPOAb (IU/ml)^a^	0.00–60.00	172.19 ± 39.16	44.57 ± 9.01	<0.01
TGAb (IU/ml) ^a^	0.00–60.00	760.45 ± 1125.21	23.85 ± 9.72	<0.01
TRAb (IU/L) ^a^	0.00–1.50	20.11 ± 11.06	0.51 ± 0.24	<0.01
ALT (U/L) ^b^	5–35	26.70 (20.30–35.78)	12.70 (11.45–14.40)	<0.01
AST (U/L) ^b^	10–67	28.50 (23.10–33.48)	24.50 (21.85–28.25)	<0.05
GGT (U/L) ^b^	7–32	20.95 (14.10–33.73)	10.40 (8.80–11.60)	<0.01
ALP (U/L) ^b^	0–500	295 (253–333)	209 (180–234)	<0.01
TBIL (μmol/l) ^b^	3.40–17.10	8.80 (6.78–13.58)	8.40 (6.40–11.65)	NS
DBIL (μmol/l) ^b^	0.00–10.00	3.61 (2.63–5.28)	2.70 (2.40–3.59)	<0.05
IBIL (μmol/l) ^b^	0.00–17.00	5.15 (4.02–7.70)	5.90 (3.95–8.44)	NS
TP (g/l) ^a^	60.0–83.0	66.03 ± 4.55	70.17 ± 4.02	NS
TG (mmol/l) ^b^	0.00–1.70	0.83 (0.58–1.23)	0.65 (0.49–0.85)	<0.05
TCHOL (mmol/l) ^a^	0.00–5.20	3.17 ± 0.52	4.54 ± 0.89	<0.05

GD, Graves’ disease; Wt, weight; FT3, free triiodothyronine; FT4, free thyroxine; TSH, thyroid stimulating hormone; TT3, total triiodothyronine; TT4, total thyroxine; TPOAb, thyroid peroxidase antibody; TGAb, thyroglobulin antibody; TRAb, thyroid stimulating hormone receptor antibodies; ALT, alanine aminotransferase; AST, aspartate aminotransferase; GGT, gamma-glutamyl transpeptidase; ALP, alkaline phosphatase; TBIL, total bilirubin; DBIL, direct bilirubin; IBIL, indirect bilirubin; TP, total protein; Tg, thyroglobulin; TCHOL, total cholesterol; NS, Not Significant.

a The data were normally distributed.

b The data were not normally distributed.

### Sample Collection

Data of thyroid function test [including total thyroxine (TT4), total triiodothyronine (TT3), free thyroxine (FT4), free triiodothyronine (FT3), and thyroid stimulating hormone (TSH), and thyroid autoantibodies [including thyroid peroxidase antibody (TPOAb), thyroglobulin antibody (TGAb), and thyroid stimulating hormone receptor antibodies (TRAb)] were collected from the laboratories of our hospital.

Blood samples for small molecule environmental substances, perfluorinated compounds [including heptafluorobutyric acid (PFBA), perfluoroheptanoic acid (PFHpA), perfluorooctanoic acid (PFOA), perfluorononanoic acid (PFNA), perfluorohexane sulfonate (PFHxS), and perfluorooctane sulphonate (PFOS)], total iodine, and SNPs analysis were taken after 10–12 h night fasting. Blood was obtained from an antecubital venous catheter and placed on ice. Serum was separated within 20 min and stored at −80°C until analysis. The methods for perfluorinated compounds and total iodine analysis were included in the **Supplementary Material**.

### Small Molecule Environmental Substances Analyzed by UHPLC–QTOF/MS

Samples were thawed at 4°C on ice. Then 100 μl sample was extracted by adding 400 μl of extraction solvent (V methanol: V acetonitrile = 1:1, containing internal standard 2 μg/ml), vortexing for 30 s, sonicating for 10 min at 4°C, and then incubating for 1 h at −20°C. The precipitated protein was then centrifuged at 4°C and 12,000 rpm for 15 min. Subsequently, 425 μl supernatant was dried in a vacuum concentrator without heating, resolved by 100 μl extraction solvent (V acetonitrile: V water = 1:1), vortexed for 30 s, sonicated for 10 min at 4°C, and centrifuged for 15 min at 12,000rpm, 4°C. Then the supernatant (60 μl) was transferred into a LC/MS vial for UHPLC-QTOF/MS analysis. To ensure data quality, 10 μl supernatant from different individual serum samples was pooled as quality control sample.

The procedures were performed following a previous study (11). In brief, LC-MS/MS analyses were performed using a 1290 UHPLC system (Agilent Technologies, Santa Clara, CA, USA) with a UPLC BEH Amide column (1.7 μm, 2.1 * 100 mm, Waters) coupled to Triple time-of-flight 6600 (Q-TOF, AB Sciex, Framingham, MA, USA). The injection volume for each sample was 1 μl. The mass spectroscopy (MS) data were collected from m/z 50–1,200 Da. The MS spectra acquisition was performed using Analyst TF 1.7 software (AB Sciex) based on the information-dependent basis (IDA) mode. In each cycle, 12 precursor ions whose intensity was greater than 100 were chosen for fragmentation at collision energy (CE) of 30 eV (15 MS/MS events per 50 ms of product ion accumulation time). The electrospray ionization (ESI) source conditions were set as following: nebulizer pressure, 60 psi; auxiliary pressure, 60 psi; curtain gas, 35 psi; source temperature 650°C ion spray voltage floating (ISVF) 5,000 V or −4,000 V in positive or negative modes, respectively.

### Multiplex PCR and SNP Analysis

A total of 29 SNPs from eight genes reported most relevant to GD in the Asian population were filtered out *via* extensive literature review. Multiplex PCR and SNP analyses were done as previously described (12). Briefly, DNA was extracted from peripheral whole blood from both GD and control groups, using a blood DNA extraction kit (Tiangen, China). Gene polymorphism typing was performed using a SNaPshot Multiplex Kit (Thermo Fisher Scientific, Applied Biosystems, Foster City, CA). Then primers used are listed in **Supplementary Table 1**.

### Statistical Analysis

The UHPLC–QTOF/MS data analysis was performed as previously described (11). Briefly, MS raw data (.wiff) files were converted to the mzXML format using Proteo Wizard and processed by R package XCMS (version 3.2). The preprocessing results generated a data matrix that consisted of the retention time (RT), massto–charge ratio (m/z) values, and peak intensity. R package CAMERA was used for peak annotation after XCMS data processing. In-house MS2 database was applied in environmental substance identification. The SIMCA 14.1 software package (Unetrics, Umea, Sweden) was used to analyze the substances. Both principal component analysis (PCA) and orthogonal partial least squared-discriminant analysis (OPLS-DA) were used for the multivariate data analysis (MVDA). The SPSS 25.0 software (SPSS Inc., Chicago, IL, USA) was used to determine significant differences between GD and normal control groups. The environmental substances with both variable importance in projection (VIP) value >1.2 and fold change (FC) >1.2 or <0.83 in the OPLA-DA model and values *P <*0.05 were considered to be significantly different. Moreover, multiple comparison methods corrected for Benjamini–Hochberg false discovery rate was conducted in response to reviewer’s suggestions, and substances with Q-values >0.05 were excluded. Binary logistic regression was first used to analyze the correlations between differential substances and GD onset. After exclusion of substances with Exp(B) value extremely high or near 1, the remaining four substances were analyzed *via* multinomial logistic regression to identify the most relevant substances with GD onset. The correlations between substances and thyroid function, as well as autoantibodies, were analyzed *via* Spearman rank correlation, and *P <*0.05 was considered as statistically significant.

The concentrations of perfluorinated compounds were calculated using calibration curves. The signal-to-noise ratios (S/Ns) were used to determine the lower limits of detection (LLODs) and lower limits of quantitation (LLOQs). The LLODs ranged from 0.20 to 3.13 nmol/L; the LLOQs ranged from 0.39 to 6.25 nmol/L for all the analytes. Correlation coefficients (R2) of regression fitting were above 0.9956 for all the analytes, indicating a good quantitative relationship between the MS responses and the analyte concentrations, which was satisfying for targeted metabolomics analysis. The recoveries determined were 88.7–100.0% for all the analytes, with all the (relative standard deviations) RSDs below 4.7%. The concentrations of perfluorinated compounds were compared between two groups, and *P <*0.05 was considered to be significantly different.

When comparing quantitative variables between the two groups, for normally distributed data, Student’s t-test was used and the results were expressed as means ± standard deviations. For data not normally distributed, Mann–Whitney *U*-test was used, and the results were expressed as medians (25th–75th percentiles).

The differences of genotypes between the two groups were analyzed by χ^2^ or Fisher’s exact methods. *P <*0.05 was considered statistically significant.

## Results

### Differential Small Molecule Environmental Substances Between GD and Control Groups

Using the UHPLC–QTOF/MS analysis, we found a total of 16 small molecule environmental substances different between GD and control groups with VIP >1.2, *P <*0.05, and Q <0.05. In the positive-ion mode (**Table 2**), six small molecule environmental substances were detected significantly different between the two groups. Compared to the controls, the levels of flavone and d-lyxose were significantly lower in the GD group. Moreover, the levels of 1-naphthol, trans-3-coumaric, famciclovir, and glutaraldehyde were significantly higher than those in the controls. Totally, 10 small molecule environmental substances were detected significantly different between the two groups under the negative-ion mode (**Table 2**). The levels of p-cresol and confertifoline were significantly lower in the GD group than those in the controls. Furthermore, compared to controls, the levels of 5,6,7,8-tetrahydro-2-naphthoic acid, nitrofurantoin, alpha-mangostin, muramic acid, ponasterone A, perseitol, benzoic acid, and 7-methylxanthine in the GD group were significantly higher.

**Table 2:** Differential environmental substances between GD and control groups.

Small molecule environmental disruptors	VIP	P	FC	Q
Benzoic acid^b^	1.20	<0.001	10.13	0.018
ponasterone A^b^	2.62	<0.001	2.35	0.0026
alpha-Mangostin^b^	2.23	<0.001	2.17	0.016
trans-3-Coumaric acid ^a^	2.88	<0.001	1.77	1.1282E-08
Glutaraldehyde^a^	2.18	<0.001	1.73	0.0009
1-Naphthol^a^	1.73	0.01	1.51	0.034
Famciclovir^a^	1.78	<0.001	1.49	0.014
5,6,7,8-tetrahydro-2-Naphthoic Acid^b^	1.87	<0.001	1.37	0.011
Nitrofurantoin^b^	2.06	<0.001	1.27	0.019
7-Methylxanthine^b^	2.16	<0.001	1.25	0.009
Muramic acid^b^	1.74	<0.001	1.21	0.016
Perseitol^b^	1.54	<0.001	1.21	0.0037
D-lyxose^a^	1.49	0.01	0.70	0.037
Confertifoline^b^	2.33	<0.001	0.66	0.0028
Flavone^a^	2.61	<0.001	0.43	0.0097
p-Cresol^b^	2.63	<0.001	0.37	0.0052

VIP, variable importance in the projection of OPLS-DA model; FC, fold change of GD group compared to control group; LOG_FC, the logarithm of FC to the base 2.

p value is from the Student’s t test; ^a^Positive-ion mode; ^b^Negative-ion mode.

We used binary logistic regression to analyze the correlation between the differential substances and GD onset (**Table 3**). After exclusion of substances with Exp(B) value extremely high or near 1, we included the remaining four substances (including confertifoline, d-lyxose, flavone, and ponasterone A) in a multinomial logistic regression and identified ponasterone A and confertifoline as the substances most relevant to GD onset (*p* = 0.007 and Exp(B) = 14.14 for ponasterone A; p = 0.002 and Exp(B) = 0.001 for confertifoline, **Table 4**).

**Table 3:** The univariate logistic analysis of the relationship between environmental substances and GD onset.

Environmental substances	B	SE	*p* Value	Exp(B)
Benzoic acid	0.14	0.16	0.38	1.146
ponasterone A	2.89	0.89	0.001	18.065
alpha-Mangostin	33.97	9.67	0.000	5.68E + 14
trans-3-Coumaric acid	69.90	19.34	0	2.27E + 30
Glutaraldehyde	30.86	8.89	0.001	2.53619E + 13
1-Naphthol	144.05	59.07	0.015	3.65E + 62
Famciclovir	83.70	29.68	0.005	2.23E + 36
5,6,7,8-tetrahydro-2-Naphthoic Acid	26.73	8.82	0.002	4.07E + 11
Nitrofurantoin	66.71	23.84	0.005	9.32E + 28
7-Methylxanthine	133.76	43.56	0.02	1.23E + 58
Muramic acid	169.25	58.95	0.004	3.20E + 73
p-Cresol	−0.19	0.06	0.001	0.825
D-Lyxose	−37.50	14.66	0.01	0
Confertifoline	−6.72	2.00	0.001	0.001
Flavone	−19.31	6.61	0.003	0
Perseitol	90.34	27.65	0.001	1.71E + 39
Iodine(µg/L)	0.04	0.01	0.000	1.042

**Table 4:** Multivariate forward stepwise logistic regression analysis the relationship between environmental substances and GD onset.

Environmental substances	B	SE	*p* Value	Exp(B)
Confertifoline	−7.39	2.40	0.002	0.001
ponasterone A	2.65	0.98	0.007	14.14
Constant	3.00	1.41	0.03	20.18

### Relationship Between Thyroid Indices and Small Molecule Environmental Substances in GD Children

There were 10 substances correlated significantly with thyroid indices in GD patients, among which seven associated with the thyroid autoantibody TPOAb (**Table 5**). The serum levels of flavone, 1-naphthol, p-cresol, confertifoline, and 5,6,7,8-tetrahydro-2-naphthoic acid were negatively correlated with the TPOAb levels, while nitrofurantoin and trans-3-coumaric acid levels were positively associated with the TPOAb levels. The thyroid function of GD children was correlated with three substances. The levels of D-lyxose correlated negatively with both the TT3 and FT4 levels. Besides, the levels of famciclovir associated positively with the FT4 levels. The concentration of 7-methylxanthine correlated negatively with the TSH and TGAb levels.

**Table 5:** Correlation between environmental substances and thyroid indexes in GD group.

Small molecule environmental substances	Related thyroid indexes	Spearman rank correlation coefficient	*p* Value
Nitrofurantoin^b^	TPOAb (IU/ml)	0.604	0.029
Famciclovir^a^	FT4 (ng/dl)	0.466	0.014
7-Methylxanthine^b^	TSH(uIU/ml)	−0.549	0.015
trans-3-Coumaric acid^a^	TPOAb (IU/ml)	−0.560	0.046
D-Lyxose^a^	FT4 (ng/dl)	−0.563	0.002
D-Lyxose^a^	TT3 (ng/ml)	−0.605	0.005
p-Cresol^b^	TPOAb (IU/ml)	−0.610	0.027
Confertifoline^b^	TPOAb (IU/ml)	−0.621	0.024
Flavone^a^	TPOAb (IU/ml)	−0.643	0.018
1-Naphthol^a^	TPOAb (IU/ml)	−0.692	0.009
7-Methylxanthine^b^	TGAB(IU/ml)	−0.770	0.009
5,6,7,8-tetrahydro-2-Naphthoic Acid^b^	TPOAb (IU/ml)	−0.786	0.001

a Positive-ion mode.

b Negative-ion mode.

### Total Iodine Levels in the GD and Control Groups

Using the ICP-MS analysis, we found that the total iodine levels in the GD group were significantly higher than those in the controls (115.9(78.70–143.28)µg/L *vs* 70.02(55.18–83.25)µg/L, *P* < 0.01). We also found that serum total iodine levels were significantly associated with GD, but only with a low Exp(B) value 1.04 *via* binary logistic regression analysis. Besides, in the GD group, total iodine levels correlated significantly with TRAb and FT4 levels, with Spearman rank correlation coefficient of 0.44 and 0.56, respectively.

Since iodine is a major environmental factor affecting GD pathogenesis, we tested total iodine levels in GD patients and used a stepwise linear regression method to exclude the impact of total iodine on the relationships between other environmental substances and thyroid indices. We found that all these relationships were independent of total iodine levels.

### Perfluorinated Compounds in the GD and Control Groups

We explored six types of perfluorinated compounds in the serum of GD and control groups, but only PFOA, PFNA, PFHxS, and PFOS were detectable. The PFBA and PFHpA serum levels were under the detection limits in all participants. No significant difference of the four perfluorinated compounds has been found between GD children and normal controls (**Table 6**).

**Table 6:** Perfluorinated compounds between GD and control groups.

Perfluorinated compounds	GD group (nmol/L)	Control group (nmol/L)	*p* Value
PFOA	0.46 (0.37–0.56)	0.45 (0.37–0.51)	0.554
PFHxS	0.28 (0.11–0.39)	0.30 (0.14–0.40)	0.564
PFNA	0.09 (0.06–0.11)	0.086 (0.06–0.11)	0.451
PFOS	0.17 (0.10–0.24)	0.17 (0.11–0.21)	0.953

GD, Graves’ disease; PFOA, perfluorooctanoic acid; PFNA, perfluorononanoic acid, PFHxS, perfluorohexane sulfonate; PFOS, perfluorooctane sulphonate.

All the data were not normally distributed, and the results were expressed as medians (25th–75th percentiles).

### Differences in the Distributions of SNP Haplotypes

A total of 29 SNPs from eight genes reported most relevant to GD in the Asian population were analyzed both in GD and control groups. We found that the distribution of rs1883832 in *CD40* gene and rs2284722 in *TSHR* gene were statistically different between the two groups (**Table 7**). GD children have higher frequencies of rs1883832-C and lower frequencies of rs2284722-A compared to controls.

**Table 7:** SNPs showing associations with GD.

Gene	SNPs	Alleles/genotypes	Case group (n = 30)	Control group (n = 30)	χ2/Fisher	P
CD40	rs1883832	C	46 (0.77)	32 (0.53)	7.18	0.007
		T	14 (0.23)	28 (0.47)		
		CC	16 (0.53)	10 (0.33)	11.42	0.011
		CT	14 (0.47)	12 (0.40)		
		TT	0 (0.00)	8 (0.27)		
TSHR	rs2284722	A	20 (0.33)	36 (0.60)	8.57	0.03
		G	40 (0.67)	24 (0.40)		
		AA	3 (0.10)	6 (0.20)	2.86	0.68
		AG	14 (0.47)	12 (0.40)		
		GG	13 (0.43)	12 (0.40)		

## Discussion

The pathogenesis of GD is affected by both genetic and non-genetic factors (5). Previous epidemiological studies have revealed multiple environmental factors influencing the incidence of GD in different populations (5), but the impact of small molecule environmental substances on the onset and progression of GD has not yet been explored. In the present study, we found 16 differentially abundant small molecule environmental substances between the two groups, among which ponasterone A is a potential risk factor, while confertifoline is a potential protective factor against GD. To explore the clinical relevance of these substances, we further evaluated the correlation between thyroid indices and the environmental substances. Our result showed the concentration of seven substances were associated with the serum levels of TPOAb, three with thyroid function and one with TGAb levels. All these relationships were independent of the total serum iodine levels. Besides, the perfluorinated compound levels, a group of newly reported TDCs, were not different between GD children and controls. Moreover, no known SNPs were found predisposing GD in this study.

The 16 differential environmental substances detected in this study can be approximately divided into three categories. The first category contains artificial synthesized compounds or their metabolites, including drugs (cortisone, famciclovir, and nitrofurantoin) and industrial chemicals (glutaraldehyde, p-cresol, 5,6,7,8-tetrahydro-2-naphthoic acid, and 1-naphthol). The second category includes chemicals of natural resources, such as flavone, confertifoline, alpha-mangostin, 3-prenyl-4-hydroxyacetophenone, muramic acid, ponasterone A, perseitol, benzoic acid, and 7-methylxanthine. The third category is gut microflora metabolites including d-lyxose, and trans-3-coumaric acid. Some of these environmental compounds have Exp(B) values near 1, which implies their minor effect on GD pathogenesis (**Table 3**). Others have very huge Exp(B) value, which may be attributed to the large standard deviation or relatively small sample size. Only one substance, ponasterone A, which is an ecdysteroids found in crustacean (13) and podocarpus macrophyllus (14), was revealed as a new risk factor of GD. The present study was carried out in Suzhou, which is a city with plenty of rivers and lakes. People here are used to consume large amounts of shrimps and crabs in their daily diet. Although the relationship between ponasterone A and AITD has not been reported before, other aquatic product consumption, such as swordfish, has been suggested as a risk factor of postpartum thyroiditis (15, 16). Future studies with much larger sample size are needed to confirm the correlation between ponasterone A and the onset of GD. Besides, confertifoline was suggested as a protective factor of GD. Confertifoline has been isolated from *Polygonum hydropiper* L. leaves and shown with antimicrobial activity (17). Further studies are needed to confirm the relationship between confertifoine and GD.

Flavonoids are a group of compounds containing two phenolic benzene rings linked by a heterocyclic pyrone or pyran ring containing an oxygen atom. Flavonoids belong to the polyphenols or plant phenolics and are contained in a variety of fruits and vegetables. Flavones are a class of flavonoids, which are rich in celery, parsley, black olives, and artichoke, *etc.* Flavones have multiple beneficial biological effects, including anti-virus, anti-inflammation, anti-allergy, and anti-oxidation. Moreover, flavones have been revealed to regulate the synthesis and secretion of thyroid hormone. Sartelet et al. reported that apigenin and luteolin, two common types of flavones, could inhibit the bioactivity of TPO. In alloxan-induced diabetic mice model, luteolin has been found to elevate the thyroid hormone levels, while in normal mice, luteolin decreased thyroid hormone levels. Isoflavone, another member of flavonoids, is a type of phytoestrogen and has been shown positively correlated with TSH levels in females. In our study, we found that flavone levels were significantly lower in GD children compared to controls, which implies that the flavone may be consumed in GD children to suppress the thyroid hormone production. Furthermore, we found that the flavone levels in GD group were negatively correlated with TPOAb levels. GD is characterized by increased oxidative stress (OS). Animals and human studies have suggested the direct contribution of ROS to the severity of clinical manifestations of GD, and antioxidants treatment improved the clinical symptoms in GD patients (18). TPOAb has been proposed as a good indicator of OS in GD children (19). Thus, the negative correlation between TPOAb and flavone levels may be explained by the anti-oxidative activity of flavone. Further studies are needed to confirm the anti-oxidative effect and potential beneficial effects of flavone in GD patients.

Trans-3-coumaric acid is also called m-coumaric acid, which belongs to the hydroxycinnamic acids. There are totally three isomers of hydroxycinnamic acid, namely p-coumaric acid, o-coumaric acid, and m-coumaric acid, among which p-coumaric acid is most common in nature and can be supplemented directly from foods. Although rich in quite a few plants, such as olives, corns, and beers, m-coumaric acid is a metabolite of caffeic acid and needs special esterase of microflora to be released into human gut (20). *In vitro*, m-coumaric acid has been reported to inhibit the proliferation of 3T3-L1 preadipocytes *via* antioxidative activity (21). However, m-coumaric acid has been suggested as the weakest antioxidant among hydroxycinnamic acids (22, 23). Although oral supplementation of p-coumaric acid had significant goitrogenic effect in rat models (24), the impact of m-coumaric acid on thyroid has not been studied. In the present study, we found that p-coumaric acid levels were not different between the two groups, while m-coumaric acid levels were significantly higher in GD groups, compared to those in controls. Since m-coumaric acid is the metabolite of gut microflora and GD can alter the gut microbial composition (25), we suggest that the higher m-coumaric acid levels in GD patients may be caused by gut flora imbalance. Further studies are needed to reveal the effect of m-coumaric acid in the pathogenesis of GD.

As a metabolite of the insecticides carbaryl and naphthalene, 1-naphthol can be used as a biomarker of exposure to these two insecticides (26). Carbaryl and naphthalene have both been reported to suppress thyroid hormone production in fishes (27, 28). *In vitro* studies have shown that carbaryl and 1-naphthol inhibited the beta-1 thyroid hormone receptor-mediated transcription. In adults, epidemiological studies refused the relevance of carbaryl and thyroid disease (29, 30). In normal children and adolescence, an association was identified between urinary 1-naphthol and TSH levels (31). In our study, serum 1-naphthol levels were significantly higher in GD group than those in controls. Further, animal studies are needed to elucidate the roles of 1-naphthol in the pathogenesis of GD.

Nitrofurantoin is an antibiotic commonly used to treat urinary tract infections and was identified as the major compound in many worldwide drug residue violations (32). Nitrofurantoin has been reported to induce immune-mediated lung and liver disease (33), sub-acute cutaneous lupus erythematosus (34), and antineutrophilcytoplasmic antibodies (ANCA)-associated vasculitis (35) in some rare cases. Nitrofurantoin can induce both humoral and cellular immune responses, increasing the T helper/T-suppressor lymphocytes ratio, and stimulating the production of autoantibodies (36, 37). In this study, we found significantly higher nitrofurantoin levels in GD children, and the levels of nitrofurantoin were positively correlated with TPOAb levels in GD patients. Further studies are necessary to elucidate the potential adverse effect of nitrofurantoin exposure on the immune pathogenesis of GD.

Genetic factors have been reported to play a major role in the etiology of GD. To investigate how the genetic factors work interactively with the environmental factors found in the present study, we analyzed 29 known SNPs from eight genes related to GD in the Asian population. Surprisingly, we did not find any SNP in GD children intensifying their GD risk. The frequency of the SNP rs1883832 in *CD40* gene with protective effect against GD in the Asian population (38) is significantly higher in GD children. Moreover, the frequency of the SNP rs2284722 in *TSHR* gene, which increases GD risk in Asians (39), was found statistically lower in GD children. It may suggest that GD children had genetic risk factors different from adults, but studies with larger sample size are needed before the conclusion can be drawn.

## Conclusion

GD is a complex disorder influenced by both genetic and environmental factors (40). Apart from previously established environmental factors, there is still a wide range of substances with correlation to GD pathogenesis. In this study, we identified several novel factors that are potentially involved in the pediatric GD pathogenesis, but further studies with larger sample size are necessary to confirm these possibilities.

## Data Availability Statement

The original contributions presented in the study are included in the article/**Supplementary Material**. Further inquiries can be directed to the corresponding author.

## Ethics Statement

The studies involving human participants were reviewed and approved by The medical ethics committee of Children’s Hospital of Soochow University. Written informed consent to participate in this study was provided by the participants’ legal guardian/next of kin.

## Author Contributions

Conceived and designed the experiments: TC. Performed the experiments: QX, XX, WG, KF, XC, RX, DZ, HW, HS, FW, and LC. Analyzed the data: QX, LC, and TC. Wrote the paper: TC, QX, LC, and JL. All authors contributed to the article and approved the submitted version.

## Funding

This study was supported by the National Natural Science Foundation of China (project code 81700793), a Suzhou Personnel Planning Project (project code GSWS2019051 and GSWS2020046) and a Suzhou Science and Technology Development Project (SS202064) awarded to TC. The study was also supported by the National Natural Science Foundation of China (project code 31701251) awarded to JL. This study was also supported by the Department of Pediatrics Clinical Center of Suzhou (Szzx201504).

## Conflict of Interest

The authors declare that the research was conducted in the absence of any commercial or financial relationships that could be construed as a potential conflict of interest.
