# Supplementary material for: Identification of Novel Environmental Substances Relevant to Pediatric Graves’ Disease
Source: Front Endocrinol (Lausanne). 2021 Jun 23;12:691326. doi: 10.3389/fendo.2021.691326 (PMC8261246; doi:10.3389/fendo.2021.691326)
Supplement: Supplementary file 1 [file DataSheet_1.docx]

Supplementary Material

*Total Iodine analysis*

Serum levels of total iodine were determined by ICP-MS (NexION^TM^ 300X, PerkinElmer) as previously described [14]. Briefly, sample transport to nebulizer was realized by a peristaltic pump at a flow rate of 3.5ml/min. The nebulizer gas was He. Iodine was determined at *m/z*=126.9, tellurium at *m/z*=129.9. 0.25% TMAH containing 40μg/L Te was used as internal standard solution. For testing quantification based on an external calibration curve different iodine concentrations were measured including 0, 0.4μg/L, 1.0μg/L, 2.0μg/L, 10μg/L, 20μg/L. The lower detection limit of total iodine was 0.097μg/L.

*Perfluorinated compounds analyzed by UHPLC-MRM-MS*

After the addition of 700 *μ*L of acetonitrile, 300 *μ*L aliquot of each sample was vortexed for 30 s, followed by incubation at -20 ^o^C overnight, and centrifugation at 12000 rpm and 4 °C for 15 min. Then 360 *μ*L supernatant was dried under a gentle nitrogen flow, and the residual was reconstructed with 108 *μ*L of extraction solution (V acetonitrile: V methanol: Vwater = 2:2:1), centrifuged at 12000 rpm and 4 °C for 15 min. An 80 *μ*L supernatant was transferred to an auto-sampler vial for LC-MS/MS analysis.

The LC-MS/MS analysis was carried out using a 1290 UHPLC system (Agilent Technologies, Santa Clara, CA, USA) with a UPLC HSS T3 column (1.8 *μ*m, 2.1×100 mm, Waters). The mobile phase A and B were 0.1% formic acid in water and acetonitrile, respectively. The column temperature was set at 35 ^o^C. The auto-sampler temperature was set at 4 ^o^C and the injection volume was 1 *μ*L. An Agilent 6460 triple quadrupole mass spectrometer (Agilent Technologies) with an AJS electrospray ionization (AJS-ESI) interface, was applied for assay development. Typical ion source parameters included: capillary voltage, +4000/-3500 V; Nozzle Voltage, +500/-500 V; gas (N_2_) temperature = 300 ^o^C, gas (N_2_) flow = 5 L/min, sheath gas (N_2_) temperature = 250 ^o^C, sheath gas flow = 11 L/min, nebulizer = 45 psi.

The multiple reaction monitoring (MRM) parameters for each of the targeted analytes were optimized by flow injection analysis. The MRM scan mode was applied to optimize the collision energy for each Q1/Q3 pair of the most sensitive transitions, and the Q1/Q3 pairs with the highest sensitivity and selectivity were selected as ‘quantifier’ for quantitative monitoring. The additional transitions acted as ‘qualifier’ for the purpose of verifying the identity of the target analytes. The MRM data was acquired and processed by Agilent Mass Hunter Work Station Software (B.08.00, Agilent Technologies). The lower limits of detection (LLODs) and lower limits of quantitation (LLOQs) were defined as the analyte concentrations that led to peaks with signal-to-noise ratios (S/N) of 3 and 10, respectively.
